# A mediation approach to understanding socio-economic inequalities in maternal health-seeking behaviours in Egypt

**DOI:** 10.1186/s12913-014-0652-8

**Published:** 2015-01-21

**Authors:** Lenka Benova, Oona MR Campbell, George B Ploubidis

**Affiliations:** Faculty of Epidemiology and Population Health, London School of Hygiene and Tropical Medicine, Keppel Street, London, WC1E 7HT UK; Centre for Longitudinal Studies, Institute of Education, London, WC1H 0AL UK

**Keywords:** Maternal health, Egypt, Socio-economic inequalities, Antenatal care, Facility delivery, Mediation analysis, Health-seeking behaviour, Care utilisation

## Abstract

**Background:**

The levels and origins of socio-economic inequalities in health-seeking behaviours in Egypt are poorly understood. This paper assesses the levels of health-seeking behaviours related to maternal care (antenatal care [ANC] and facility delivery) and their accumulation during pregnancy and childbirth. Secondly, it explores the mechanisms underlying the association between socio-economic position (SEP) and maternal health-seeking behaviours. Thirdly, it examines the effectiveness of targeting of free public ANC and delivery care.

**Methods:**

Data from the 2008 Demographic and Health Survey were used to capture two latent constructs of SEP: individual socio-cultural capital and household-level economic capital. These variables were entered into an adjusted mediation model, predicting twelve dimensions of maternal health-seeking; including any ANC, private ANC, first ANC visit in first trimester, regular ANC (four or more visits during pregnancy), facility delivery, and private delivery. ANC and delivery care costs were examined separately by provider type (public or private).

**Results:**

While 74.2% of women with a birth in the 5-year recall period obtained any ANC and 72.4% delivered in a facility, only 48.8% obtained the complete maternal care package (timely and regular facility-based ANC as well as facility delivery) for their most recent live birth. Both socio-cultural capital and economic capital were independently positively associated with receiving any ANC and delivering in a facility. The strongest direct effect of socio-cultural capital was seen in models predicting private provider use of both ANC and delivery. Despite substantial proportions of women using public providers reporting receipt of free care (ANC: 38%, delivery: 24%), this free-of-charge public care was not effectively targeted to women with lowest economic resources.

**Conclusions:**

Socio-cultural capital is the primary mechanism leading to inequalities in maternal health-seeking in Egypt. Future studies should therefore examine the objective and perceived quality of care from different types of providers. Improvements in the targeting of free public care could help reduce the existing SEP-based inequalities in maternal care coverage in the short term.

**Electronic supplementary material:**

The online version of this article (doi:10.1186/s12913-014-0652-8) contains supplementary material, which is available to authorized users.

## Background

Health-seeking behaviours comprise one of the direct pathways leading to the widely reported association between socio-economic position (SEP) and health outcomes [1]. Understanding the mechanisms underlying this association is crucial to devising effective interventions to reduce avoidable and unfair inequalities in health outcomes. Inequities in the coverage of maternal care interventions have gained prominence in light of Millennium Development Goal efforts to reduce maternal and neonatal mortality by 2015 and beyond [2]. In addition to deaths, maternal near-miss events and other complications resulting in morbidity and long-term disability also carry devastating effects on the lives of women, children and families in the form of physical, psychological and socio-economic sequelae [3-5]. Antenatal care (ANC) and delivery care prevent maternal and perinatal deaths [6], but their coverage relies on numerous complex factors such as availability, quality and cost of care, as well as their utilisation by women.

Egypt witnessed large decreases in maternal mortality in the last two decades; a decrease from 174 to 84 per 100,000 live births between 1992–3 and 2000 [7] and a further decline to 66 by 2010 [8]. This reduction was most likely achieved through a combination of increasing ANC coverage, skilled birth attendance, improved quality of care, access to emergency obstetric care and fertility-reducing socio-economic development, in particular women’s education [9]. Yet, in the five years before 2008, 78% of births to women with complete secondary or higher education were preceded by four or more ANC visits, but only 45% of births among women with no education were [10]. Physical access does not appear to present barriers to accessing care as 95% of Egypt’s population live within 5 km from the nearest health facility [11] and only 4% of maternal deaths in the 2000 maternal mortality survey were classified as avoidable due to long distance to reach a hospital [7]. However, the existence of health care facilities may not necessarily translate into care which is available, acceptable, affordable and good quality. Substandard care and referral delays were implicated as the second most important preventable causes of maternal mortality in 2000 [7,12]. The proportion of facility deliveries occurring in public facilities has steadily declined from 63% in 1992 to 27% in 2008 [10]. This trend toward increasing private care utilisation may be a result of perceived and/or real quality of care deficits in the public sector [13].

Socio-economic resources are well-established determinants of maternal care utilisation in low and middle-income countries [14,15]. In Egypt, important gaps in the understanding of the extent of socio-economic inequalities in maternal health-seeking behaviours remain [16]. Specifically, no study has presented an adjusted analysis of the association between SEP and maternal health-seeking behaviour on a nationally-representative sample. Each separate dimension of maternal health-seeking behaviour (e.g., timing, intensity and costs of care) may exhibit different direction and magnitude of association with SEP. A detailed understanding of the association between SEP and the separate dimensions of health-seeking behaviour is required.

This study uses the most recent nationally-representative survey Demographic and Health Survey (DHS) conducted in Egypt in 2008 to address its three objectives. Firstly, we aim to assess the levels of health-seeking behaviours related to maternal care and their accumulation throughout the process of health-seeking leading toward receipt of the complete maternal care package. Understanding whether the current inequalities in maternal health-seeking behaviours are a result of knowledge-related preferences or differences in access to financial resources is essential to designing effective interventions aimed at their elimination. Therefore, our second objective involved exploring the mechanisms underlying the association between SEP and maternal health-seeking behaviours. For this purpose, latent variables capturing the socio-cultural capital and economic capital aspects of SEP were constructed. We specified an adjusted mediation model to assess the direct, indirect (mediated by economic capital) and total (direct plus indirect) effects of socio-cultural capital on maternal health-seeking behaviours [17,18]. This innovative approach allowed not only a quantification of the association between the two dimensions of SEP and health-seeking behaviour outcomes in adjusted analysis, but also an assessment of their relative importance as drivers of inequalities. Lastly, we examine the effectiveness of targeting of free public ANC and free public delivery care.

## Methods

### Study sample

The analysis is based on a nationally-representative survey of ever-married women aged 15–49 from the 2008 Egypt DHS. To examine health-seeking behaviours related to maternal care, we assessed behaviours surrounding the most recent birth among women who reported having given birth in the five years preceding the survey. We analysed costs of care among the subsample of women whose most recent birth occurred in the twelve months prior to survey to limit the need for women to recall costs over longer periods of time. The average annual inflation rate in consumer prices in the period between 2003 and 2007 was 7.5% [19].

### Ethics

The collection of the DHS data was approved by local authorities in Egypt; respondents’ informed consent was sought. This secondary analysis of anonymised data was approved by the Research Ethics Committee of the London School of Hygiene and Tropical Medicine, UK.

### Measures of SEP

#### Socio-cultural capital

Education and literacy capture knowledge, ability to access new information, cognitive skills, previous exposure to authority, ability to interact with modern institutions such as healthcare providers, and have been linked to effective negotiation within familial power structures [20-23]. The education level of other decision-making members of the household influence health-seeking decisions through awareness of the benefits of medical assistance during pregnancy and support in seeking care [24]. Employment status captures the utilisation of attained education and exposure to wider social networks through workplace interactions. The latent measure of socio-cultural capital was based on woman’s and her husband’s education (continuous variable reflecting number of years of education) and woman’s literacy (illiterate, reads/writes with difficulty or reads/writes easily). Husband’s occupational category (not employed, unskilled manual, skilled manual, services, agriculturally employed, agriculturally self-employed, sales, clerical and professional) was used. A binary variable captured the working status of the female respondents, as the large majority (87.1%) reported not to be working. High scores on the latent variable represented higher socio-cultural capital.

#### Economic capital

Household-level material resources available to meet the direct and indirect costs of care were captured by the economic capital latent variable [25]. This construct would ideally be captured by measures such as income, consumption or expenditure. However, the collection and post-processing of such measures is resource-intensive and requires sophisticated econometric techniques. The DHS wealth index provides a more stable measurement of household-level resources than consumption expenditure [26], although the underlying constructs may not coincide [27]. A household wealth index score based on principal component analysis of 79 separate household-level variables was constructed in the DHS. In order to be able to replicate the current analysis on other datasets collected in Egypt with fewer available variables, we constructed a simpler variable to reflect the relative distribution of accumulated resources among households in which women who have had a birth in the five-year recall period resided. Its ten variables consisted of binary descriptive characteristics of the current living residence: utilities (water piped into dwelling, flush toilet), household ownership of assets (fridge, car, mobile, colour TV, water heater, automatic washing machine), ownership of a bank account, and level of crowding. Crowding was calculated as the number of household members per bedroom, and dichotomized as being above or below the median level (1.5 members per bedroom) within the sample of women. High scores on the latent variable index of economic capital represented wealthier households.

### Health-seeking behaviour outcomes

#### Antenatal care

**Figure 1:**
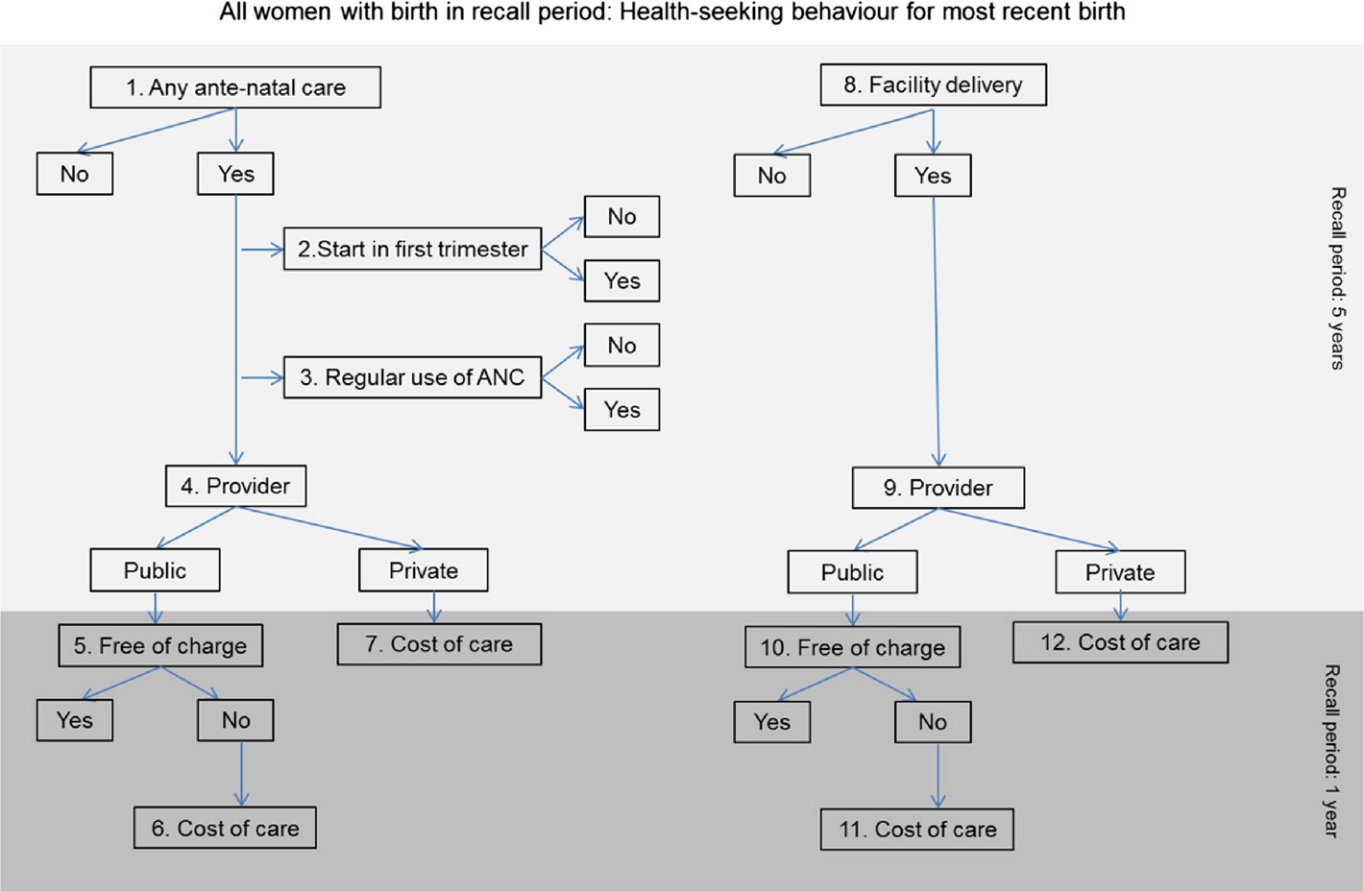
**Dimensions of maternal health-seeking behaviour.**

Seven dimensions of ANC utilisation for the most recent pregnancy were assessed (Figure 1). A binary variable indicated whether the woman received any facility-based ANC during the pregnancy. If ANC was utilised, binary variables described its timeliness (whether first ANC visit occurred in first trimester of pregnancy), intensity (four or more ANC visits were received during pregnancy), and the type of provider used (public or private). The definition of private provider included any facility-based non-public providers, such as private hospitals, clinics, doctors, the Egyptian Family Planning Association, the Clinical Services Improvement project, and other non-governmental organisation/private providers. Only 2.1% of women who used ANC reported receiving care from a combination of public and private providers; we grouped women who used both public and private providers with those who solely used private providers.

#### Delivery care

We used five health-seeking behaviours to describe women’s utilisation of delivery care (Figure 1). Firstly, a binary variable captured whether the most recent delivery in the five-year recall period occurred in a health facility. Among the subset of women with facility deliveries, we examined the use of private providers. A binary categorisation of private providers was constructed, combining all non-public sector providers (private hospitals/clinics, private doctor’s offices and other private medical facilities, including non-governmental organisations).

### Cost of care

The analysis of price of antenatal and delivery care was limited to births occurring in the 12 month period before survey. Among women who used public providers, we analysed the binary outcome capturing whether this care was obtained free of charge. Among paying users of public services and women who used private providers, we analysed the amount paid for care (Figure 1). Specifically, women were asked whether they paid for ANC services (excluding laboratory or medication costs) separately during each visit, on a one-time basis, or received ANC for free. Among paying ANC users, we created a variable capturing the per-visit cost of ANC. In order to arrive at the per-visit cost among women who incurred one-time payments, the total ANC expenditure was divided by the number of ANC visits during pregnancy. For women who reported paying for each ANC visit separately, the amount reported paid for the last ANC visit during pregnancy was used. The cost of delivery service (excluding laboratory and medication expenses), reported by women with a facility-based birth was analysed. We constructed a binary variable capturing whether delivery care was received for free or not. Among women who reported paying for delivery care, a continuous variable captured the amount paid. The resulting continuous variables reflecting price of ANC and delivery care in Egyptian pounds (EGP), 1USD = 5.5 EGP in 2008), which were estimated separately by provider type.

#### Complete maternal care package

For the purposes of analysing the receipt of the basic elements of maternal care, we defined a complete maternal care package as the receipt of timely (first visit in the first trimester of pregnancy) and regular (four or more ANC visits during pregnancy) facility-based ANC and facility delivery. Women who did not receive any or all of these three care elements were considered not to have received the complete package. This binary classification was made regardless of whether such care was obtained from public or private providers and irrespective of the cost incurred for this care.

### Confounders

We identified *a priori* confounders of the association between socio-cultural capital, economic capital and maternal health-seeking behaviours [14], including woman’s age group at the time of the most recent birth, parity group and whether pregnancy under analysis was intended or not [28]. Elements of availability of health services were captured in the residence variable (urban or rural) and whether respondent had unmet need for contraception at the time of the survey [29]. We created a binary variable for female head of household to capture the extent of the respondent’s autonomous decision-making. Additional variables related to maternal care were also used in the analysis of subsequent health-seeking outcomes, including the use of any ANC, use of regular ANC, use of private ANC, receipt of information about delivery complications during pregnancy, and delivery by caesarean section.

### Statistical analysis

Latent variable modelling is an approach to quantify unobservable constructs by utilising common variance among observed indicators. Variance that is not common, including random error, is disregarded from the latent summary. The aim is to reduce the dimensionality of the observed data, but to retain a good representation within the latent variable identified [30,31]. Latent variables capturing socio-cultural and economic capital were constructed in Mplus/v.7.11 using the Weighted Least Squares, Mean and Variance adjusted (WLSMV) estimator. Factor loadings of each observed variable represent the association between this indicator and the underlying construct. Proportion of missing data in the observed variables in both latent constructs was minimal, and all observations were included. Model fit was assessed with the Comparative Fit Index (CFI), the Tucker Lewis Index (TLI) and the Root Mean Square Error of Approximation (RMSEA). The latent scores were standardised to a mean of zero and standard deviation of one.

**Figure 2:**
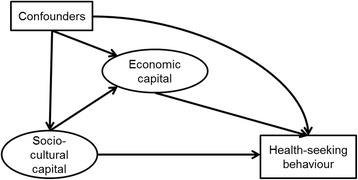
**Conceptual path diagram of the structural model.**

Figure 2 shows the conceptual framework of the analysis in which socio-cultural capital can be directly or indirectly (through economic capital) associated with the outcomes. Continuous latent scores for both variables were entered in the mediation model, in order to jointly estimate their associations. The direct effects of both measures of SEP on binary outcomes was modelled in logistic regression and odds ratio was the main effect estimate. The total effect of socio-cultural capital (sum of its direct and indirect effects) on binary outcomes is expressed as the sum of changes in the probability of outcome (*ΣΔp)*.

We estimated the mean price of ANC and delivery care by provider type and assessed the effectiveness of free public care targeting by comparing the mean socio-cultural and economic capital scores between women who received ANC or delivery care free of charge at public facilities with those who used these public services but paid for care. The extent of SEP inequalities accumulated throughout the health-seeking process for the most recent birth was estimated by comparing the mean SEP scores of women who received the complete maternal care package with those who received no maternal care. Inequalities in this multidimensional outcome and in samples used for assessment of targeting were examined in the subsample of women who delivered in the twelve month period preceding the survey, using the t-test.

We accounted for the complex survey sampling (clustering, stratification and weights) by using the *svyset* command in Stata in the descriptive overview of the sample and in analysis of targeting and multidimensional outcomes. The Stata *medeff* command was used for mediation analysis, incorporating robust standard errors adjusting for clustering and sampling weights [32]. The proportion of missing data in the majority of the outcome variables was minimal and we utilised complete case analysis in the mediation analysis.

## Results

The latent measurement models for both SEP constructs had an acceptable fit to the data; the RMSEA level was ≤0.05 and the CFI/TFI ≥0.972 (Additional file 1). The median standardised socio-cultural capital score was 0.139 (inter-quartile range [IQR]: −0.575 to 0.501) and median economic capital score was −0.058 (IQR: −0.380 to 0.491). An observation with a median socio-cultural capital score was described as a woman with six years of education, with difficulty reading and writing, not currently in employment, with a husband self-employed in agriculture who achieved 12 years of education. The median economic capital score described a household which owned a fridge, a mobile phone, a colour TV, had a piped water connection, but did not own a car, a water heater, an automatic washing machine, a flush toilet in the dwelling, a bank account, and in which crowding was less than the national median of 1.5 persons per bedroom.

Table 1 displays the demographic and socio-economic characteristics of the samples analysed in this study. The differences between the overall sample of women with a birth in five years preceding the survey (sample A) and the subsample of women who delivered in the 12-month period before the survey (sample B) included a younger age distribution in sample B, as well as lower parity, lower proportion of unwanted pregnancies, higher proportion of deliveries by caesarean section, lower proportion of women with female head of household status and higher mean socio-cultural capital score. Among ANC users, women with non-missing cost information did not differ from the women with missing price of care data in the distribution of demographic, pregnancy-related or SEP factors. The proportion of missing data in price paid for care did not differ between users of private and public providers in ANC or delivery samples (*X*^2^ test p-value 0.786 and 0.258, respectively).

**Table 1:** **Distribution of demographic, socio-economic and delivery-related variables in study samples**

**Characteristics**	**Sample of women**	**All women with live birth in recall period**		**ANC users**			**Facility delivery users**		
				**All**		**ANC cost available**	**All**		**Delivery cost available**
	***Recall period***	***5 years***	***1 year***	***5 years***	***1 year***	***1 year***	***5 years***	***1 year***	***1 year***
	***Sample name***	***A***	***B***	***C***	***D***	***E***	***F***	***G***	***H***
	***Sample size***	***7,896***	***2,581***	***5,861***	***2,058***	***1,994***	***5,715***	***1,962***	***1,724***
Age group	14-19 (%)	9.2	10.9	9.4	10.8	10.7	9.3	11.0	10.9
	20-24	32.5	35.6	33.5	36.2	36.1	31.8	35.6	35.5
	25-29	30.6	29.8	31.1	30.4	30.4	31.2	30.0	29.9
	30-34	16.6	14.7	15.7	14.0	14.1	16.4	14.3	14.1
	35-39	8.7	7.5	8.2	7.3	7.3	8.9	7.7	8.0
	40-49	2.4	1.5	2.1	1.3	1.4	2.4	1.4	1.6
	*X* ^2^ *p value*	*<0.001*			0.824*			0.750*	
Parity	1 (%)	26.6	34.0	30.6	37.4	37.3	30.6	38.4	37.9
	2	28.1	26.9	28.8	26.5	26.4	29.0	26.9	27.8
	3	21.6	19.8	21.2	19.2	19.4	21.2	19.1	18.9
	4 or more	23.7	19.3	19.4	16.9	16.9	19.2	15.6	15.4
	*X* ^2^ *p value*	*<0.001*			0.254*			0.219*	
Household status	Female head (%)	78.0	73.6	79.3	74.5	74.6	79.7	74.7	73.9
	*X* ^2^ *p value*	*<0.001*			0.241*			0.033*	
Desire for pregnancy	Unwanted (%)	15.3	14.0	13.7	12.1	12.3	13.7	12.3	12.8
	Wanted	84.7	86.0	86.3	87.9	87.7	86.3	87.7	87.8
	Missing	<0.1	<0.1	<0.1	0.0	<0.1	<0.1	0.0	<0.1
	*X* ^2^ *p value*	*0.003*			0.182*			0.110*	
Need for contraception	Unmet (%)	12.2	12.7	11.3	11.8	11.8	11.4	12.7	12.8
	*X* ^2^ *p value*	*0.428*			0.811*			0.695*	
Region	Urban (%)	38.2	37.4	43.8	41.8	41.5	45.3	43.0	43.6
	Rural	61.8	62.6	56.2	58.2	58.5	54.7	57.0	56.4
	*X* ^2^ *p value*	*0.419*			0.066*			0.189*	
C-section delivery	Yes (%)	29.2	31.5	34.3	35.5	35.4	40.3	41.5	42.7
	*X* ^2^ *p value*	*0.007*			0.744*			0.005*	
Socio-cultural capital	Mean	0.027	0.061	0.137	0.134	0.132	0.138	0.146	0.152
	(SE)	(0.0118)	(0.0158)	(0.0123)	(0.0166)	(0.0165)	(0.0128)	(0.0172)	(0.0178)
	*T test p value*	*0.001*			0.155**			0.142**	
Economic capital	Mean	0.064	0.063	0.177	0.148	0.145	0.198	0.175	0.177
	(SE)	(0.0133)	(0.0169)	(0.0139)	(0.0178)	(0.0172)	(0.0144)	(0.0184)	(0.0195)
	*T test p value*	*0.968*			0.106**			0.379**	

SE: standard error. Complex survey design (weighting, clustering and stratification) was accounted for in calculations of proportions and sample sizes reported.

*Testing the hypothesis that users of ANC/facility delivery services in the last year before survey who had missing data in the variable for cost of those services were drawn from the same population as users with available cost information.

**T test p value testing that the difference in mean SEP scores between samples of women is 0.

**Figure 3:**
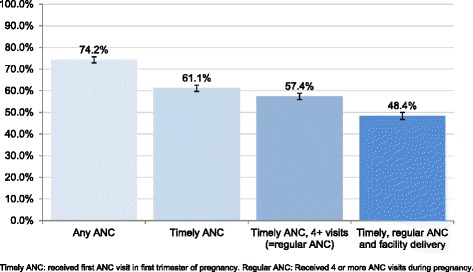
**Proportions of women with a birth in five years preceding the survey receiving elements of the complete maternal health package.**

Levels of the twelve maternal health-seeking behaviours are described in Table 2. Among women with a birth in the five years preceding the survey, 74.2% reported having received ANC for their most recent birth. Within users of ANC, 82.5% received ANC starting in the first trimester, 90.6% received regular ANC, and 76.6% visited a private provider. In terms of delivery care, 72.4% of women reported having delivered in a health facility, 63.0% of them in a private facility. Figure 3 shows that when the combination of ANC and delivery care is assessed, 48.4% (95%CI 46.7%-50.0%) of women with a birth in five years preceding the survey obtained the complete maternal care package for their most recent birth.

**Table 2:** **Maternal health-seeking behaviours among ever-married women for most recent birth**

**Health-seeking behaviour outcome**		**Variable type**	**Samples and missing data**				**Distribution of outcome in analysed sample and 95% CI**
			**Eligible sample and recall period**	**Eligible sample (size)**	**Missing data (%)**	**Analysed sample (size)**	
*Antenatal care (ANC)*							
**1.**	**Used ANC**	Binary	All women with birth 5 years prior to survey	7,896	-	7,896	74.2% (72.8 - 75.6)
**2.**	**ANC in 1** ^**st**^ **trimester of pregnancy**	Binary	All women with birth 5 years prior to survey who used any ANC	5,861	0.2	5,851	82.5% (81.3 - 83.7)
**3.**	**Regular use of ANC (4+ visits)**	Binary	All women with birth 5 years prior to survey who used any ANC	5,861	0.9	5,798	90.6% (89.7 - 91.4)
**4.**	**ANC from private provider**	Binary	All women with birth 5 years prior to survey who used any ANC	5,861	-	5,861	76.6% (74.9 - 78.1)
**5.**	**Public provider: ANC free of charge**	Binary	All women with birth 1 year prior to survey who used public ANC	426	1.6	419	38.1% (32.7 - 43.7)
**6.**	**Public provider: Cost of ANC (EGP)**	Continuous	All women with birth 1 year prior to survey who used public ANC and paid for care	259	-	259	GM 2.0 (1.7 - 2.3)
**7.**	**Private provider: Cost of ANC (EGP)**	Continuous	All women with birth 1 year prior to survey who used private ANC and paid for care	1,633	3.6	1,575	GM 18.4 (17.8 – 19.1)
*Delivery care*							
**8.**	**Delivered in a health facility**	Binary	All women with birth 5 years prior to survey	7,896	<0.1	7,893	72.4% (70.8 - 73.9)
**9.**	**Used private delivery facility**	Binary	All women with birth 5 years prior to survey who delivered in a health facility	5,715	-	5,715	63.0% (61.1 - 64.9)
**10.**	**Public provider: Delivery free of charge**	Binary	All women with birth 1 year prior to survey who delivered in a public health facility	700	12.7	611	24.1% (20.3 – 28.3)
**11.**	**Public provider: Cost of delivery (EGP)**	Continuous	All women with birth 1 year prior to survey who delivered in a public health facility and paid for delivery	464	-	464	GM 96.6 (83.5 – 111.7)
**12.**	**Private provider: Cost of delivery (EGP)**	Continuous	All women with birth 1 year prior to survey who delivered in a private health facility and paid for delivery	1,262	11.8	1,113	GM 489.8 (462.5 – 518.7)

Complex survey design was accounted for in calculations of proportions, means and confidence intervals. EGP: Egyptian pound 95%CI: 95% confidence interval GM: Geometric mean.

Among women who had a birth in the twelve months before the survey, 38.1% of those attending public providers reported receiving free ANC; 24.1% of women who delivered in a public facility reported receiving care free of charge (Table 2). The mean reported cost of a paid ANC visit was 2.0 EGP among user of public and 18.4 EGP among users of private providers. The mean cost of public delivery services was 97 EGP, differing between caesarean section deliveries (203 EGP) and normal deliveries (64 EGP). The mean price of private facility delivery was 490 EGP; 889 EGP for a caesarean section delivery and 300 EGP for a normal delivery.

### Mediation analysis

The result of adjusted analysis of the association between the two latent variables and any ANC use (Table 3) shows that a one unit increase in socio-cultural capital was associated with 1.55 higher odds of any ANC (95%CI 1.40-1.72) and with 1.79 higher odds of private ANC use (95%CI 1.57-2.04). Higher socio-cultural capital scores were marginally associated with higher odds of receiving ANC in the first trimester (OR = 1.13, p-value 0.096) and regular use of ANC (OR = 1.20, p-value 0.037). The total effect of socio-cultural capital on the four binary ANC outcomes was significant and positive, and the strength of the direct association between economic capital and these outcomes was larger than the direct association of socio-cultural capital. The associations between socio-cultural capital and any ANC use and socio-cultural capital and private ANC use were mainly the result of its direct effect (42% and 31% were mediated by economic capital, respectively). On the other hand, the associations between socio-cultural capital and first trimester ANC use and socio-cultural capital and regular ANC use were largely mediated through economic capital (indirect effect accounted for 73% and 66% of the total, respectively). Both socio-cultural and economic capital strongly predicted facility use for delivery care. The direct effect of a one unit increase in socio-cultural capital was associated with 1.31 higher odds of delivering in a facility (95%CI 1.16-1.47) and with 1.51 higher odds of delivering in a private facility (95%CI 1.34-1.70). We estimated that economic capital mediated 52% of the total effect of socio-cultural capital on facility delivery and 35% of its effect on private facility use.

**Table 3:** **Adjusted effects of socio-cultural capital and economic capital on binary maternal health-seeking behaviours**

**Utilisation of maternal services**	**(1) Direct effect of socio-cultural capital**	**(2) Direct effect of economic capital**	**(3) Total effect of socio-cultural capital**	**(4) % of total effect of socio-cultural capital mediated by economic capital**
	***OR (95% CI)***	***OR (95% CI)***	***ΣΔp (95% CI)***	***% (95% CI)***
**Any ANC use** ^**1**^	1.55 (1.40 to 1.72)	2.18 (1.92 to 2.48)	0.10 (0.09 to 0.11)	42% (38% to 48%)
**ANC in first trimester** ^**1**^	1.13 (0.98 to 1.30)	2.08 (1.75 to 2.46)	0.05 (0.04 to 0.07)	73% (57% to 100%)
**Regular use of ANC** ^**2**^	1.20 (1.01 to 1.42)	2.31 (1.85 to 2.89)	0.03 (0.02 to 0.04)	66% (53% to 90%)
**Private ANC use** ^**1**^	1.79 (1.57 to 2.04)	1.91 (1.61 to 2.26)	0.12 (0.10 to 0.13)	31% (27% to 35%)
**Facility delivery use** ^**3**^	1.31 (1.16 to 1.47)	2.12 (1.84 to 2.45)	0.08 (0.06 to 0.09)	52% (44% to 65%)
**Private delivery facility** ^**4**^	1.51 (1.34 to 1.70)	1.80 (1.55 to 2.11)	0.13 (0.11 to 0.15)	35% (30% to 42%)
**Public ANC free of charge** ^5*^	2.08 (1.39 to 3.10)	0.56 (0.32 to 0.97)	0.12 (0.04 to 0.21)	0%
**Public delivery free of charge** ^4*^	1.30 (0.88 to 1.90)	0.69 (0.43 to 1.10)	0.03 (−0.04 to 0.10)	not applicable

95%CI: 95% confidence interval. OR: Odds ratio associated with one unit increase in capital score.

ΣΔp: Total effect of socio-cultural capital was calculated as the sum of the changes in probability of outcome based on both indirect (mediated by economic capital) and direct effects.

*Free public care was assessed in subsample of women with a birth in the 12 month period preceding the survey.

^1^Adjusted for age group, parity, household status, pregnancy wanted, unmet need and region.

^2^Adjusted for age group, parity, household status, pregnancy wanted, unmet need, region, and private ANC provider use.

^3^Adjusted for age group, parity, household status, pregnancy wanted, unmet need, region, any ANC use, and information on delivery complications.

^4^Adjusted for age group, parity, household status, pregnancy wanted, unmet need, region, any ANC use, information on delivery complications, and delivery by c-section.

^5^Adjusted for age group, parity, household status, pregnancy wanted, unmet need, region, and regular ANC.

Table 3 also shows the associations between socio-economic and cultural capital and receiving public ANC and delivery services free of charge. Among women who received public ANC, a one unit increase in socio-cultural capital was associated with 2.08 higher odds of free ANC care (95%CI 1.39-3.10), but a one unit increase in economic capital halved the odds of free ANC care (OR = 0.56, 95%CI 0.32-0.97). None of its effect was therefore mediated by economic capital. Neither SEP measure was significantly associated with the odds of receiving free public delivery care; mediation analysis was therefore not applicable.

**Figure 4:**
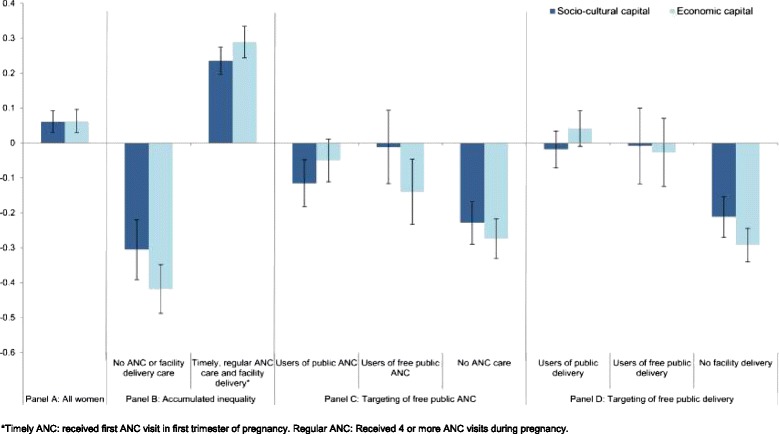
**Socio-cultural and economic capital mean scores (95% confidence intervals) within subsamples of women who gave birth in the twelve month period preceding survey.**

Figure 4 displays the mean levels of socio-economic capital and economic capital between various sub-samples of women with a birth in the year prior to survey. Panels A and B show that the mean scores among women who received complete maternal care was significantly higher than the mean score of all women in this sample and higher than among women who did not receive any facility-based maternal services. Further, Panel C contrasts the mean scores of three subsamples of women according to ANC health-seeking behaviour outcomes. The mean socio-cultural and economic capital scores among women who received free public ANC were marginally lower than among all users of public ANC (p-values 0.052 and 0.029, respectively). However, non-users of ANC had significantly lower mean socio-cultural (p < 0.001) and economic (p = 0.005) capital scores compared to women who received free public ANC. Panel D shows that the data were consistent with no difference in the mean socio-cultural capital (p = 0.983) and economic capital (p = 0.221) scores between women who received free public delivery care and all women who received public delivery care. However, the mean socio-cultural capital and economic capital scores were significantly lower among women who did not deliver in a facility compared to those who received free public care (p-values 0.002 and <0.001, respectively).

## Discussion

Our findings showed that socio-economic position was a strong determinant of maternal health-seeking behaviours in Egypt. In adjusted models, both socio-cultural and economic capital scores were significantly positively associated with receiving ANC and delivering in a health facility. Socio-cultural capital was the main driver of private provider preference, but available economic resources largely determined the timeliness and intensity of such care. Women who received free public ANC or free public delivery care were not significantly poorer than all women using public care. However, women who did not receive ANC or delivery care had significantly lower mean socio-cultural capital and economic capital scores than women accessing free public care.

The average cost of a single ANC visit was approximately ten times higher in private compared to public facilities and the average cost of delivery services was five times higher in private compared to public facilities. A woman receiving the minimum recommended ANC care (four visits) and a caesarean section delivery would be expected to pay between 211 EGP (all care from public facilities) and 970 EGP (all care from public facilities) for these services; excluding laboratory charges, medications and other costs such as transportation, child-care and foregone income. In light of the 41.2% poverty rate in Egypt in 2008–2009 [33], it is not surprising that only half of women received the three components of the complete maternal care package, and that this multidimensional health-seeking outcome was strongly socio-economically patterned.

In order to capture the most recent patterns of health-seeking behaviour, we analysed the circumstances surrounding the most recent birth in the recall period. The overall response rate to the EDHS 2008 survey was high (98.8%). However, this analysis faced several limitations. The DHS collected information on the health-seeking behaviours surrounding women’s most recent live birth in the recall period. Health-seeking behaviours of women whose most recent pregnancies resulted in a stillbirth are not represented in these data. The cross-sectional and observational design of this study limits our ability to assess causal relationships between SEP and health-seeking behaviours. In addition, the data were collected before the dramatic changes in socio-political situation in early 2011, which may have influenced the patterns of both supply and demand for care, thereby potentially limiting the generalisability of our findings [34].

The two latent SEP measures constructed and used in this study relied on observed self-reported variables, which are reliable and present lower risk of measurement error and recall bias compared to income, expenditure or consumption variables [35]. The DHS wealth index has been criticised due to inclusion of components, such as utilities or items dependent on utilities (i.e., electrical appliances), which are more prevalent in urban areas [36]. Our measure of economic capital faced similar issues. Another limitation of asset-based measures stems from inability of binary measures of ownership to capture potentially important variability in the quality of assets and their state of repair [37]. We attempted to minimise this potential source of error in our analysis by using the highest grade of asset (i.e., *colour* TV, *mobile* phone, *automatic* washing machine), but were not able to assess their functionality.

All measures of health-seeking behaviour analysed in this study were self-reported. Whereas we expect the report of the occurrence of a live birth in the recall period to be reliable, the health-seeking behaviour variables may be affected by measurement error and recall bias. The health-seeking behaviour variables (e.g., number of ANC visits, type of delivery facility) may be affected by measurement error, in particular recall bias and social desirability bias [38,39]. A study in rural China showed that validity of women’s recall of ANC timing and components up to five years since the delivery showed high sensitivity (~90%) [39]. Women’s self-report of the level of health facility utilised in delivery care carried high sensitivity and specificity in Mozambique. [40] The information about ANC was collected only about the most recent birth and although information about delivery circumstances of all births in the 5 year recall period are available, we chose to only assess both ANC and delivery health-seeking behaviours for the most recent pregnancy and delivery to minimise such error. However, the validity of women’s recall of the various dimensions of maternal health-seeking has not been assessed in Egypt, and may be differentially biased according to the time that had elapsed since the events took place [41,42].

We conducted sensitivity analysis using skilled birth attendance instead of facility delivery as a delivery care outcome and obtained similar results (not shown). We attempted to reduce recall bias by limiting the analysis of price of care to births which occurred in the twelve months prior to survey. The costs of laboratory tests and medications during ANC and delivery care were not included in the analysis due to high level of missingness. Therefore, interpretation of such partial information about pregnancy care expenditures should be cautious.

The main strength of this study stems from including both socio-cultural and economic aspects of SEP in the mediation model predicting their association with various dimensions of maternal health-seeking behaviour. This approach allowed for the estimation of the total effect of socio-cultural capital as well as decomposition into its direct and indirect components. However, for this estimated model to be valid, there should be no unmeasured confounding in any part of Figure 2 [43]. While we attempted to identify and include all potential confounders, the presence of unmeasured confounding cannot be completely ruled out. Women’s obstetric risk profile may be one such potential confounder, but the type of information (e.g. complications in previous deliveries, a complete history of assisted deliveries) which would allow the construction of such profile was not collected on the DHS. Instead, women’s age group and parity were used as proxies. Indicators capturing the supply and quality of maternal care, while not available in the dataset, may have acted as effect modifiers or potential sources of unmeasured confounding. In sensitivity analyses of the effect of region of residence as a proxy for geographical availability of services, the data were consistent with no effect modification (results not shown).

## Conclusions

Further improvements in maternal health in Egypt are highly dependent on increasing coverage of maternal interventions among the poorest and most disadvantaged segments of society [44]. To our knowledge, this is the first analysis of socio-economic inequalities in maternal health-seeking behaviours in Egypt employing a formally specified mediation framework. The results showed that socio-economic inequalities in the coverage of basic maternal health interventions exist. By analysing the effects of socio-cultural and economic resources separately, we provided insights into the mechanisms through which socio-economic position determines health-seeking behaviours. Effectiveness of free public care targeting must improve in order to reach the most socio-economically vulnerable women. In order to inform the design of effective interventions to reduce the remaining inequalities, future research should focus on quality of care and perceptions of different provider types. This approach would be particularly pertinent in light of the common occurrence of medical staff simultaneously practicing in both public and private sectors. Lastly, an exploration of other determinants of maternal care utilisation among socio-economically vulnerable women, such as their personal interaction with care providers, could help explore other enabling factors or barriers to accessing maternal care [45].

## Additional file

Additional file 1:
**Descriptive characteristics of component variables in latent SEP measurements, among sample of women who gave birth in 5 year preceding survey (n = 7,896).**

## Abbreviations

ANC: Antenatal care
CFI: Comparative fit index
DHS: Demographic and health survey
EGP: Egyptian pound
RMSEA: Root mean square error of approximation
SEP: Socio-economic position
TLI: Tucker lewis index
WLSMV: Weighted least squares, mean and variance adjusted estimator
