# Supplementary material for: A mediation approach to understanding socio-economic inequalities in maternal health-seeking behaviours in Egypt
Source: BMC Health Serv Res. 2015 Jan 21;15:1. doi: 10.1186/s12913-014-0652-8 (PMC4307186; doi:10.1186/s12913-014-0652-8)
Supplement: Additional file 1: — Descriptive characteristics of component variables in latent SEP measurements, among sample of women who gave birth in 5 year preceding survey (n = 7,896). [file 12913_2014_652_MOESM1_ESM.docx]

**Supplementary Material 1. Descriptive characteristics of component variables in latent SEP measurements, among sample of women who gave birth in 5 year preceding survey (n=7,896)**

| **Component variables** | **Distribution**  **(n=7,896)** | **Standardized factor loading (SE)** |
| --- | --- | --- |
| **Socio-cultural capital** |  |  |
| *Woman: Years of education* |  |  |
| Mean *(95% CI)* | 14.7 (9.6-19.8) | 0.74 (0.01) |
| *Woman: Literacy* |  |  |
| Illiterate *(column %)* | 27.5 | 0.71 (0.01) |
| Reads/writes with difficulty | 6.6 |  |
| Reads/writes easily | 65.8 |  |
| Missing | 0.1 |  |
| *Woman: Employment status* |  |  |
| Currently works (%) | 12.8 | 0.30 (0.02) |
| *Husband: Years of education* |  |  |
| Mean *(95% CI)* | 14.2 (9.9-18.5) | 0.89 (0.01) |
| *Husband: Occupational category* |  |  |
| Not in employment *(column %)* | 1.6 | 0.43 (0.01) |
| Unskilled manual | 6.9 |  |
| Skilled manual | 29.0 |  |
| Services | 14.3 |  |
| Agriculture, employed | 9.5 |  |
| Agriculture, self-employed | 6.4 |  |
| Sales | 3.3 |  |
| Clerical | 3.7 |  |
| Professional | 24.5 |  |
| Missing | 0.8 |  |
|  |  |  |
| **Economic capital** |  |  |
| *Household utilities* |  |  |
| Piper water in dwelling *(%)* | 90.1 | 0.46 (0.02) |
| Flush toilet *(%)* | 44.4 | 0.89 (0.01) |
| *Household asset ownership* |  |  |
| Refrigerator *(%)* | 91.4 | 0.75 (0.02) |
| Car *(%)* | 7.1 | 0.64 (0.02) |
| Mobile phone *(%)* | 41.6 | 0.54 (0.01) |
| Colour TV *(%)* | 89.0 | 0.64 (0.02) |
| Water heater *(%)* | 35.4 | 0.90 (0.01) |
| Automatic washing machine *(%)* | 18.5 | 0.91 (0.01) |
| *Household service ownership* |  |  |
| Bank account *(%)* | 7.1 | 0.61 (0.02) |
| *Household crowding* |  |  |
| <1.5 persons/bedroom *(%)* | 48.6 | 0.48 (0.01) |

95%CI: 95% confidence interval SE: Standard error

Goodness of fit criteria for latent variables

| **Latent variable** | **CFI** | **TLI** | **RMSEA (90%CI)** |
| --- | --- | --- | --- |
| Socio-cultural capital | 0.999 | 0.998 | 0.011 (0.001 - 0.020) |
| Economic capital | 0.981 | 0.972 | 0.050 (0.046 - 0.053) |

CFI - Comparative index fit (values>0.950 indicate good fit). TLI - Tucker Lewis index (values >0.950 indicate good fit). RMSEA - Root mean square error of approximation (values of <0.06 indicate good fit).
